# Absorption cross section of gold nanoparticles based on NIR laser heating and thermodynamic calculations

**DOI:** 10.1038/s41598-020-75895-9

**Published:** 2020-11-02

**Authors:** Mazen Alrahili, Viktoriia Savchuk, Kelly McNear, Anatoliy Pinchuk

**Affiliations:** 1 Department of Physics and Energy Science, University of Colorado Colorado Springs, 1420 Austin Bluffs Parkway, Colorado Springs, CO 80918 USA; 2 UCCS BioFrontiers Center, University of Colorado Colorado Springs, 1420 Austin Bluffs Parkway, Colorado Springs, CO 80918 USA; 3 Physics Department, School of Science, Taibah University, Janadah Bin Umayyah Road, Medina, 42353 Saudi Arabia

**Keywords:** Thermodynamics, Nanoparticles

## Abstract

We present a method for measuring the optical absorption cross section ($\sigma_{abs}$) of gold nanoparticles (GNPs) based on optically heating the solution of GNPs with an 808 nm near-infrared (NIR) laser and measuring the temperature increase of the solution. We rely on the theoretical calculations based on the heat diffusion equations and experimental measurements based on the energy balance equations to measure the $\sigma_{abs}$ and the temperature distribution of single GNPs. Several morphologies, including gold nanospheres (GNSs), spherical gold nanoparticle conjugate (AuNPC), which are 20 nm GNSs surface-functionalized with an IR 808 dye, gold nanorods (GNRs), and gold nanourchins (GNUs), were studied. The study found that a single 20 nm GNS has the lowest $\sigma_{abs}$ and temperature distribution as compared to 100 nm GNUs. By increasing the size of GNSs from 20 to 30 nm, the magnitude of $\sigma_{abs}$ as well as temperature distribution increases by a factor of 5. The $\sigma_{abs}$ values of 20 and 30 nm GNSs calculated by Mie theory and the experimentally measured are in a good agreement. GNRs with equivalent radius ($R_{eq}$) 9.16 nm show the second lowest $\sigma_{abs}$. By increasing the $R_{eq}$ by a factor of 2 to 19.2 nm, the measured $\sigma_{abs}$ and temperature distribution also increased by a factor of 2. We also estimated $\sigma_{abs}$ for GNUs with diameters at 80 and 100 nm, which also have higher $\sigma_{abs}$ values. This work confirms that we can use temperature to accurately measure the $\sigma_{abs}$ of a variety of GNPs in solution.

## Introduction

The remarkable optical properties of GNPs have captured a lot of attention over the last few decades due to a broad range of applications as varied as imaging, sensing, gene and drug delivery, and more recently, biomedical applications such as hyperthermia using near-infrared (NIR) light^1–5^. NIR light is often used since biological tissues such as skin have low absorption and high penetration in the first and second biological windows—700–900 nm and 1000–1700 nm, respectively^6–11^. GNPs can be developed in different morphologies such as nanospheres, nanorods, nanoshells, nanourchins, and more. Depending on the shapes and sizes, the surface plasmon resonance (SPR) can be over a wide spectrum from visible to NIR region^12,13^. For instance, GNSs have SPR in the visible region and GNRs have strong SPR in the NIR region. A methodology for synthesizing GNPs has been a challenging task in the scientific community. Therefore, different methods have been used to develop many morphologies. The best chemical method to achieve the enlarging demand of GNPs is well-known as the citrate reduction method due to many advantages including inexpensive reductant, water solvent, and non-toxic^14^. Other chemical methods can be found and described in the literature^15–18^.

Typically, optical losses in plasmonic nanoparticles (NPs) are considered to be an obstacle for applications such as plasmonic optical waveguides. However, for hyperthermia applications, optical losses are beneficial since GNPs can be used as local sources of heat conversion from light into thermal energy. Recently, we investigated a set of nano-gold with different morphologies such as GNSs, GNRs, GNUs, and gold conjugates with IR dye attached to 20-nm GNSs. We found that AuNPC exhibit an enhanced ‘light to heat energy conversion efficiency (η)’ by a factor four than those in the unmodified gold nanospheres^19^.

It is important to know the η-value as a function of a laser power density for optimizing the hyperthermia procedure as well as an efficient light source that can be used in its applications. The results can be extended to calculate the rise in the local temperature, δT_h_, per single NP of different shapes, which can be useful in translating potential candidates for photothermal therapy from benchtop to clinic. Furthermore, one can use the δT value to calculate $\sigma_{abs}$ in the selected particle. The $\sigma_{abs}$ of colloidal GNPs can be experimentally measured by using a UV–Vis absorption spectrophotometer^20^. One can also calculate the optical absorption (or extinction) cross section of the NPs by using Mie theory^21^. Typically, the experimental and theoretical results for the $\sigma_{abs}$ agree well for GNSs of different sizes. For non-spherical NPs, numerical methods such as Discrete Dipole Approximation (DDA) can be used to calculate their $\sigma_{abs}$ values^22^. It is interesting to know if the $\sigma_{abs}$ measured by using an optical spectrometer, or calculated by Mie theory, matches the values extracted from temperature measurements in hyperthermia-type or thermo-plasmonic type experiments.

In this article, we estimated the $\sigma_{abs}$ values of nanogold of a variety of nontrivial morphologies dispersed in a liquid host after selective dosages of NIR irradiations. This is done by measuring the temperature rise of nanogold in the solution when exposed to a continuous laser of an 808 nm wavelength at varying power densities and then averaging the value per particle. The obtained $\sigma_{abs}$ is compared to the calculated value in the Mie theory. It is found that the $\sigma_{abs}$ value sensitively varies in the samples of varied morphologies consistently to the calculated values. This opens a door for developing a basic understanding of the fundamental properties of nanogold of complex morphologies.

## Results

### Optical extinction spectra of nanogold of different morphologies

**Figure 1:**
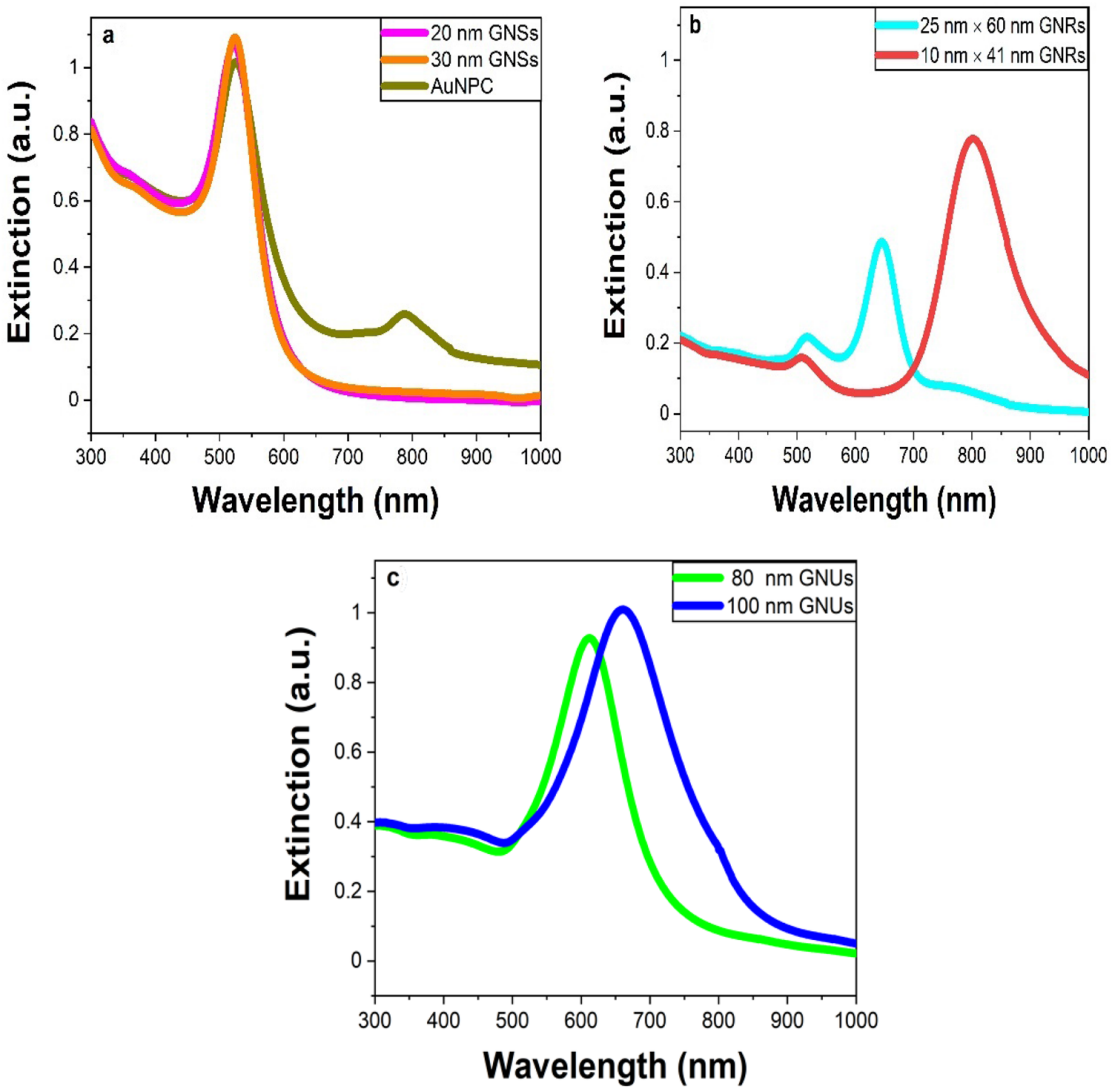
The extinction spectra of the different nanogold samples of (**a**) 20 and 30 diameters GNSs, (**b**) 25 and 10 diameters GNRs and (**c**) 80 and 100 nm diameters GNUs. All solutions were measured at a normalized optical density (OD) 1.

Here, seven different types of nanogold with various sizes were studied, including 20 and 30 nm GNSs, 20 nm core AuNPC, 80 and 100 nm GNUs, and 25 nm × 60 nm and 10 nm × 41 nm GNRs. Figure 1a shows the extinction spectra of the SPR bands obtained for these samples. 20 nm and 30 nm GNSs exhibit an intense SPR band in the visible region with a peak position at 520 nm. As mentioned above, the AuNPC sample, which is a surface-functionalized sample of 20 nm GNSs with an IR 808 dye, exhibits a strong SPR band at 520 nm with a weak satellite band at 788 nm (from the dye). In 80 nm GNUs, the SPR band is displaced at 620 nm, which is shifted to 680 nm on raising their average size to 100 nm. Further, 25 nm × 60 nm and 10 nm × 41 nm GNRs, having different diameters of 25 nm and 10 nm, respectively, exhibit two well-known SPR bands of transverse and longitudinal modes of the plasmons^7,10^. The SPR bands we observed here are very much consistent to those reported in different shapes of nanogold as given in Table S1 in the supporting information.

### Heating temperature profile of nanogold samples

**Figure 2:**
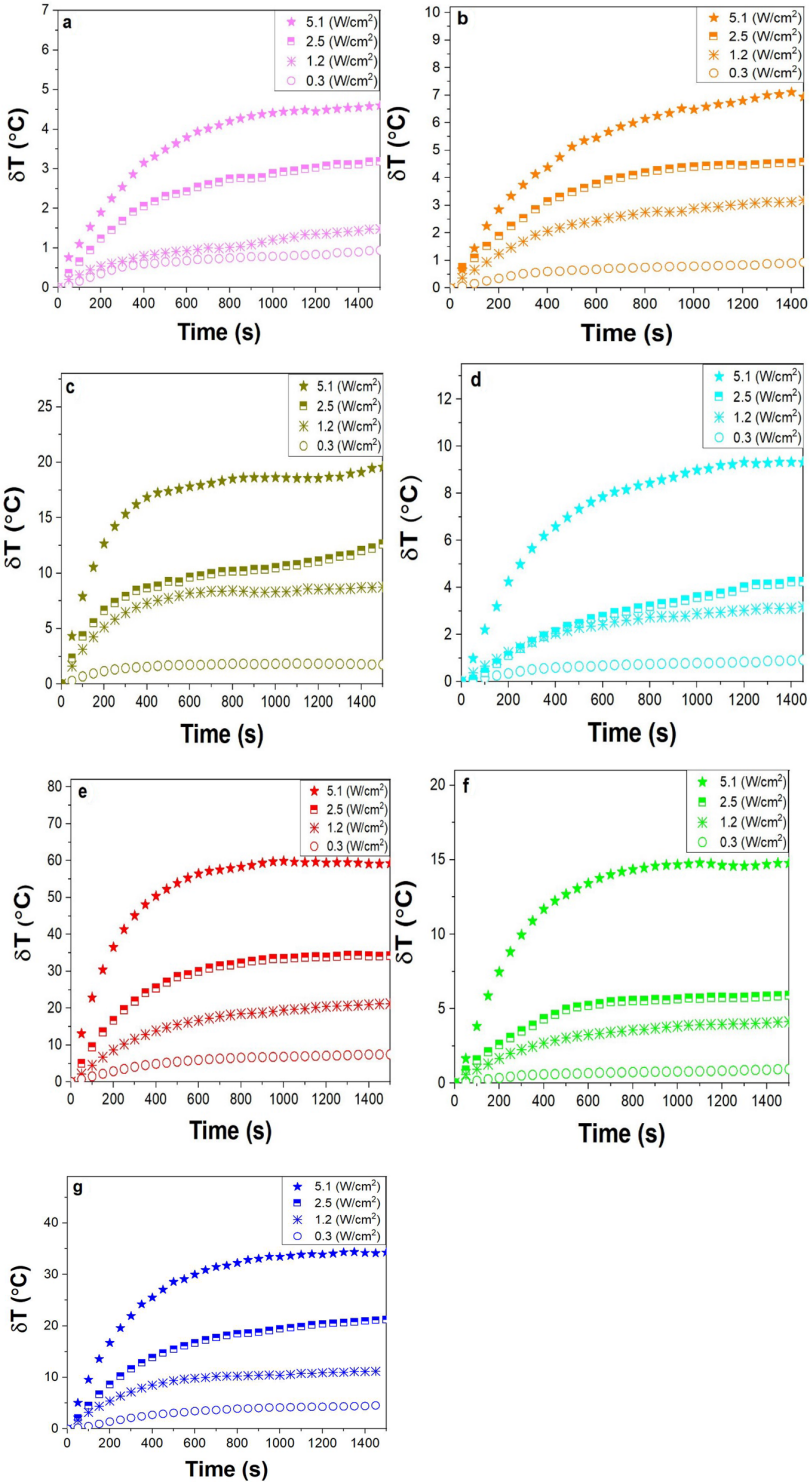
The evolution of δT as a function of irradiation time (s) for (**a**) 20 nm GNSs, (**b**) 30 nm GNSs, (**c**) AuNPC, (**d**) 25 nm × 60 nm GNRs, (**e**) 10 nm × 41 nm GNRs (**f**) 80 nm GNUs, and (**g**) 100 nm GNUs. All the solutions were measured over 25 min of irradiation by the CW NIR laser of densities of 0.3, 1.2, 2.5, and 5.1 W/cm^2^. All solutions were excited at OD = 1.

Seven colloidal suspensions of the nanogold samples were optically heated by the NIR laser at different power densities of 0.3, 1.2, 2.5, and 5.1 W/cm^2^. During laser irradiation, the nanogold absorbs part of the light which it then converts into thermal energy, which transfers to the surrounding medium. As shown in Fig. 2, the highest temperature elevation was thus observed for the 10 nm × 41 nm GNRs, with the second most value found in the 100 nm GNUs. The results confirm that the GNRs and GNUs conduct usefully larger temperature rise than the spheroids of the same volume. This heat increase is well-supported with the literature^23–25^. A value δT = 60 °C is found for the 10 nm × 41 nm GNRs in which the SPR band overlaps the laser signal and that is dropped to 35 °C in the 100 nm GNUs. In all cases, δT_h_ follows the same trend at all four power densities. The larger laser power used the larger δT_h_ value induced in the present samples.

### Temperature increase by a single nanoparticle

To describe the local temperature profile inside and around nanogold dispersed in a solution, we use a heat transfer equation^26^,

1 $$\rho C_{p} \frac{{\partial T\left( {\varvec{r}} \right)}}{\partial t} = \nabla \cdot \left[ {\kappa \nabla T\left( {\varvec{r}} \right)} \right] + q\left( {\varvec{r}} \right),$$

where $\rho$ is the density of the medium of thermal conductivity ($\kappa$) and specific-heat-capacity $\left( {C_{p} } \right)$ at constant pressure, and $T\left( {\varvec{r}} \right)$ is the absolute local temperature. In the steady-state regime, when the local temperature reaches its equilibrium profile, i.e. $\frac{{\partial T\left( {\varvec{r}} \right)}}{\partial t} = 0$, this equation reduces to,

2 $$\nabla \cdot \left[ {\kappa \nabla T\left( {\varvec{r}} \right)} \right] = - q\left( {\mathbf{r}} \right).$$

Assuming k-values for gold and water as $\kappa_{g} = 318 \;{\text{W}}\;{\text{m}}^{ - 1} {\text{K}}^{ - 1} \;\;\;{\text{and}} \;\;\;\kappa_{w} = 0.6 \;{\text{W}}\;{\text{m}}^{ - 1} {\text{K}}^{ - 1}$ respectively, Eq. (2) can be split into two parts;

3 $$\kappa_{g} \nabla^{2} {\text{T}}\left( {\varvec{r}} \right) = - q\left( {\varvec{r}} \right),\;\;{\text{inside the nanogold}}$$

4 $${\text{and}}\;\;\;\kappa_{w} \nabla^{2} {\text{T}}\left( {\varvec{r}} \right) = 0,\;\;{\text{outside the nanogold}}.$$

Mathematically, Eqs. (3) and (4) are equivalent to Poisson’s and Laplace’s equations in electrostatics, where $T$ is equivalent to the electrostatic potential, $\kappa$ is equivalent to dielectric permittivity, and $q\left( {\varvec{r}} \right)$ is equivalent to local charge density^27^. Using the analogy with electrostatics, we can find their solutions of the second-order differential equations and write $\updelta {\text{T }}_{NP} \left( {\varvec{r}} \right)$ for a GNS of radius R as,

5 $$\updelta {\text{T }}_{NP} \left( {\varvec{r}} \right) = \frac{P}{{4\pi \kappa_{w} r}},\quad{\text{for r }} > {\text{ R}}$$

and

6 $$\updelta {\text{T }}_{NP} \left( {\varvec{r}} \right) = \frac{P}{{4\pi R\kappa_{g} }},\quad{\text{for r }} < {\text{ R}},$$

where *P* is the total electromagnetic power absorbed by the nanogold, which can be calculated as;

7 $$P = \smallint q\left( {\varvec{r}} \right)d\user2{r = }\sigma_{abs} I,$$

where *I* is the power density of the incoming electromagnetic signal integrated over the surface of a spheroid. For a non-spherical morphology, thus one can rewrite an expression^27^,

8 $$\frac{P}{{{\delta T}_{NP} = 4R_{eq} \beta \kappa_{gold} }} = \frac{{\sigma_{abs} I }}{{4R_{eq} \beta \kappa_{gold} }}$$

where $R_{eq}$ is the equivalent NP radius of a volume V equal to that of a spheroid, i.e.^28,29^,

9 $$R_{eq} = \left( \frac{3V}{4} \right)^{1/3}$$

An average $\beta$ value for nanorods can be given by the following expression^30^,

10 $$\beta = { 1} + \, 0.{96587}ln^{2} \left( \frac{h}{d} \right),$$

where $h$ is the length of the GNRs of diameter $d$. In simplifying the calculations, we consider only the core diameter and ignore contribution from the dye for AuNPC and sharp branches for GNUs. Hence, both have the same (β) 1 thermal capacitance geometrical correction factor (dimensionless).

As mentioned above, when heated by laser irradiation, the colloidal sample will reach equilibrium, $\frac{{\partial T\left( {\varvec{r}} \right)}}{\partial t} = 0$. For example, Fig. 2a plots the ${\delta T}$ values obtained as a function of the laser power density for the nanogold samples of different shapes. To find an average value per particle, one needs to determine GNPs present per unit volume, i.e., N_g_ per mL, in the sample. In this experiment, we used 3 mL of the GNP solution. It can be expressed at the equilibrium as follows,

11 $$\delta T_{s} = \frac{\delta Tm}{{N }}.$$

**Figure 3:**
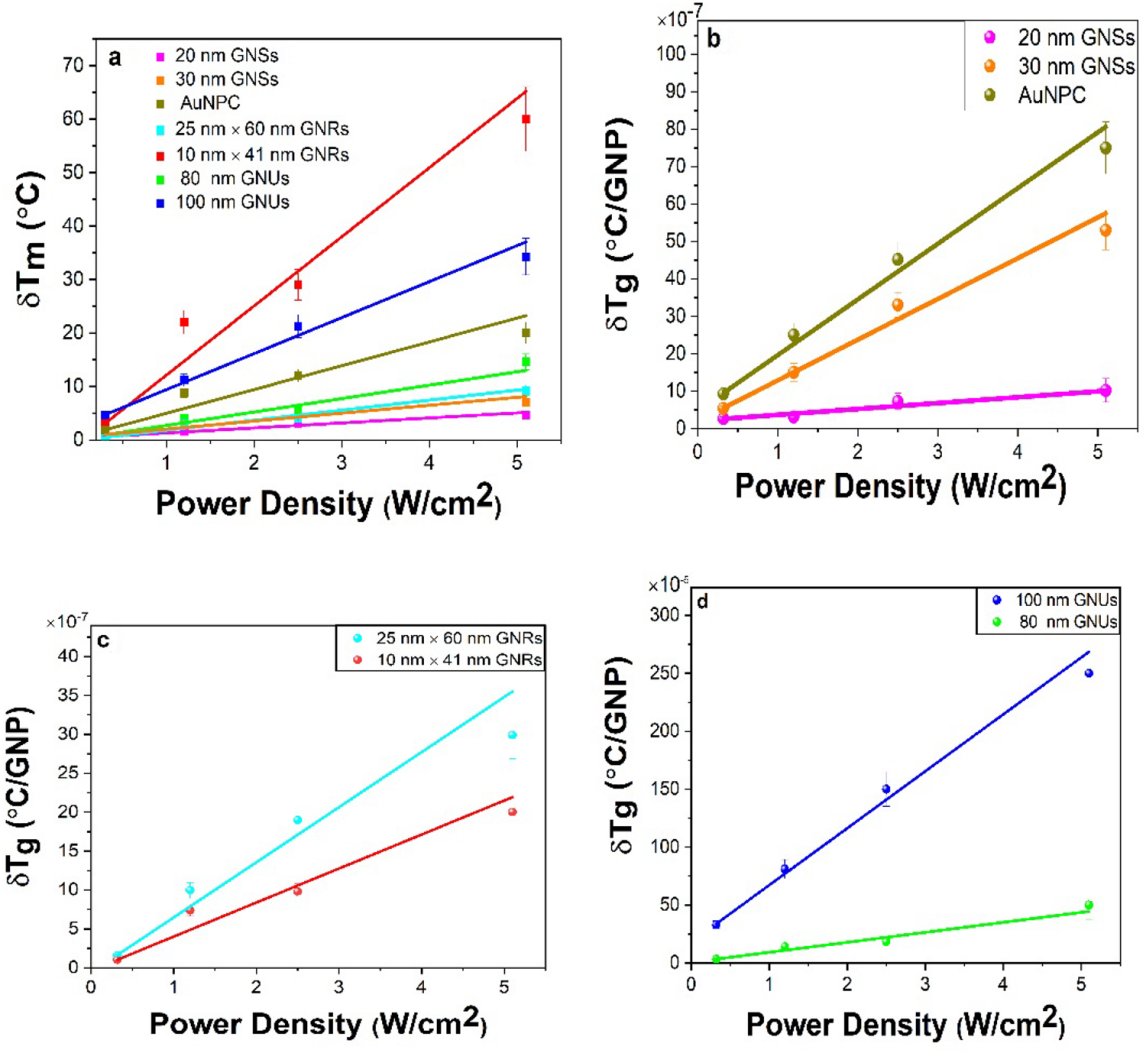
Temperature elevation in the colloidal solutions as a function of power density; (**a**) the net values on different GNPs in the solutions after subtracting the background, with average values per single GNPs for (**b**) 20 nm and 30 nm GNSs and AuNPC, (**c**) 25 nm × 60 nm and 10 nm × 41 nm GNRs, and (**d**) 80 nm and 100 nm GNUs.

Here, $\delta T_{s}$ is the saturation temperature (°C) obtained per single particle following a net δT_m_ value in Fig. 2 at the equilibrium and then graphed in Fig. 3a as a function of power density. In the experimental conditions, $N$ = N_g_V_s_, as can be seen in Table S1 in the supporting information, and V_s_ is the sample volume. It should also be noted that since the above equation determines the temperature distribution by a single GNP in solution, we need to solve the energy balance equation of the system in order to determine the temperature change of the entire solution. The energy balance of the system of temperature conservation changes in a solution due to GNPs can be written as follows^31,32^,

12 $$\mathop \sum \limits_{i} m_{i} C_{i} \frac{dT}{{dt}} = Q_{in} - Q_{out} ,$$

where $m_{i} \;\;and\;\;C_{i}$ are the mass and the specific heat of the components, respectively. $Q_{in}$ is the input heat (incident light) and $Q_{out}$ is the output heat dissipated in the surrounding medium. At the equilibrium position, $Q_{in}$ ≅ $Q_{out}$. Thus, the energy balance can be rewritten as,

13 $$m_{S} C_{S} \delta T_{S} = m_{g} C_{g} \delta T_{g}$$

where $m_{S}$ and $C_{S}$ are the mass and the specific heat of the solution and $m_{g} ,C_{g} , \;\;\;{\text{and}} \delta T_{g}$ are the mass, the specific heat, and the temperature change of the GNPs. Rearranging, we get

14 $$\delta T_{g} = \frac{{m_{S} C_{S} }}{{m_{g} C_{g} }}\delta T_{S}$$

The significance of Eq. (12) is that the mass in single GNPs plays a major role in the net temperature change. In different nanogold morphologies studied here, all the parameters are constant except for $m_{g}$ which varies depending on the size of the GNPs as given in Table S1. For each type of nanogold, the temperature change in the solution was calculated and reported in Table S2. It should be noted that $\delta T_{S}$ is calculated using Eq. (9), while $\delta Tm$ is measured from the experiments at the equilibrium as shown in Fig. 3a.

### Absorption cross section of nanogold of different morphologies

To estimate the temperature distribution of single GNPs, one must rely on the theoretical calculations and experimental measurements to determine the $\sigma_{abs}$ value, i.e. written from Eq. (5) as,

15 $$\sigma_{abs} \left( {{\text{m}}^{{2}} } \right) \, = \frac{{\delta T_{g} }}{I}4{\text{R}}\kappa_{w} = \Phi 4{\text{R}}\kappa_{w}$$

For a non-spherical nanomaterial, it can be expressed after Eq. (8) as,

16 $$\sigma_{abs} \left( {{\text{m}}^{{2}} } \right) \, = \frac{{\delta T_{g} }}{I}4{ }R_{eq} \beta \kappa_{w} \cong \Phi 4R_{eq} \beta \kappa_{w} ,$$

where Φ is the slope, °C *m^2^/W, as can be extracted from the plots in Fig. 3b–d, with ${{\updelta {\text{T}} }}_{NP} \left( {\varvec{r}} \right)$ ≅ $\delta T_{g}$. The experimental $\sigma_{abs}$ per particle is presented in Table S3 after subtracting the background, as plotted in Fig. S1. A similar value is obtained by feeding the experimental results in Eqs. (13) and (14). For spherical nanomaterials, 20 nm GNSs have a measured $\sigma_{abs}$ = (2.1 $\pm$ 0.316) × $10^{ - 18} \;{\text{m}}^{2}$, which is enhanced to (10.0 $\pm$ 0.937) × $10^{ - 18} {\text{m}}^{2}$ in the 30 nm GNSs.

In the case of GNRs, we obtained the experimental $\sigma_{abs}$ values by feeding the experimental results into Eq. (14). Thus, 25 nm × 60 nm GNRs, having R_eq_ = 19.2 nm and β = 1.74, as determined by Eqs. (7) and (8), respectively, have $\sigma_{abs}$ = (16.2 $\pm$ 1.676) × $10^{ - 18}$ ${\text{m}}^{2}$. A bit smaller $\sigma_{abs}$ = (7.49 $\pm$ 1.254) × $10^{ - 18}$ ${\text{m}}^{2}$ persists in the sample of 10 nm × 41 nm GNRs with R_eq_ = 9.16 nm and β = 2.92. The same procedure yields $\sigma_{abs}$ = (25.5 $\pm$ 4.084) × $10^{ - 16}$ ${\text{m}}^{2}$ for 80 nm GNUs, which is promoted to (18.4 $\pm 1.216$) × $10^{ - 15} {\text{m}}^{2}$ on an increased core diameter to 100 nm.

## Discussion

Eventually, a tailored $\sigma_{abs} {\text{value}},{\text{ which is }}$ markedly varied in nanogold of its effective size is varied in three representative morphologies, viz., spheroids, rods, and urchins, displays five nontrivial effects of its (i) surface structure, (ii) local symmetry, (iii) charge carrier density of surface plasmons, (iv) polarization of surface charges, and (v) interactions (with the surroundings) on its optical properties in a nanocolloid (or a nanofluid) as follows. For example, an induced temperature in a sample of single 20 nm GNSs after laser irradiation (at a 5.1 W/cm^2^ power) is found to be δT_g_ = 1.02 × $10^{ - 6}$ °C as seen in Fig. 3b in a model relation in Eq. (13), with $\sigma_{abs}$  = (2.1 $\pm$ 0.316) × $10^{ - 18} m^{2}$. When the diameter was increased to 30 nm, this value is increased nearly five times; with a final $5 \times 10^{ - 6}$ °C value in the same conditions, due to prominent effects of the first four factors. Consistently, the $\sigma_{abs}$ value is also increased by a similar factor, $\sigma_{abs}$ = (10.0 $\pm$ 0.937) × $10^{ - 18}$ ${\text{m}}^{2}$. A ${\text{value of}} \sigma_{abs}$ = 2.4 × $10^{ - 18}$ ${\text{m}}^{2}$ is calculated by Mie theory for 20 nm GNSs, while 8.5 × $10^{ - 18}$ ${\text{m}}^{2}$ for 30 nm GNSs, very similar to the measured $\sigma_{abs}$ values. Further, the AuNPC sample is especially noteworthy in regards to increasing the $\sigma_{abs}$ because it not only has a larger hydrodynamic diameter—due to a surface functionalizing with dye molecules at the surfaces^11^, but it also has an absorbance in resonance with the laser light used for the irradiation. As a result, it enhances the ${{ \upsigma }}_{{{\text{abs}}}}$ value significantly over the unfunctionalized GNSs.

**Figure 4:**
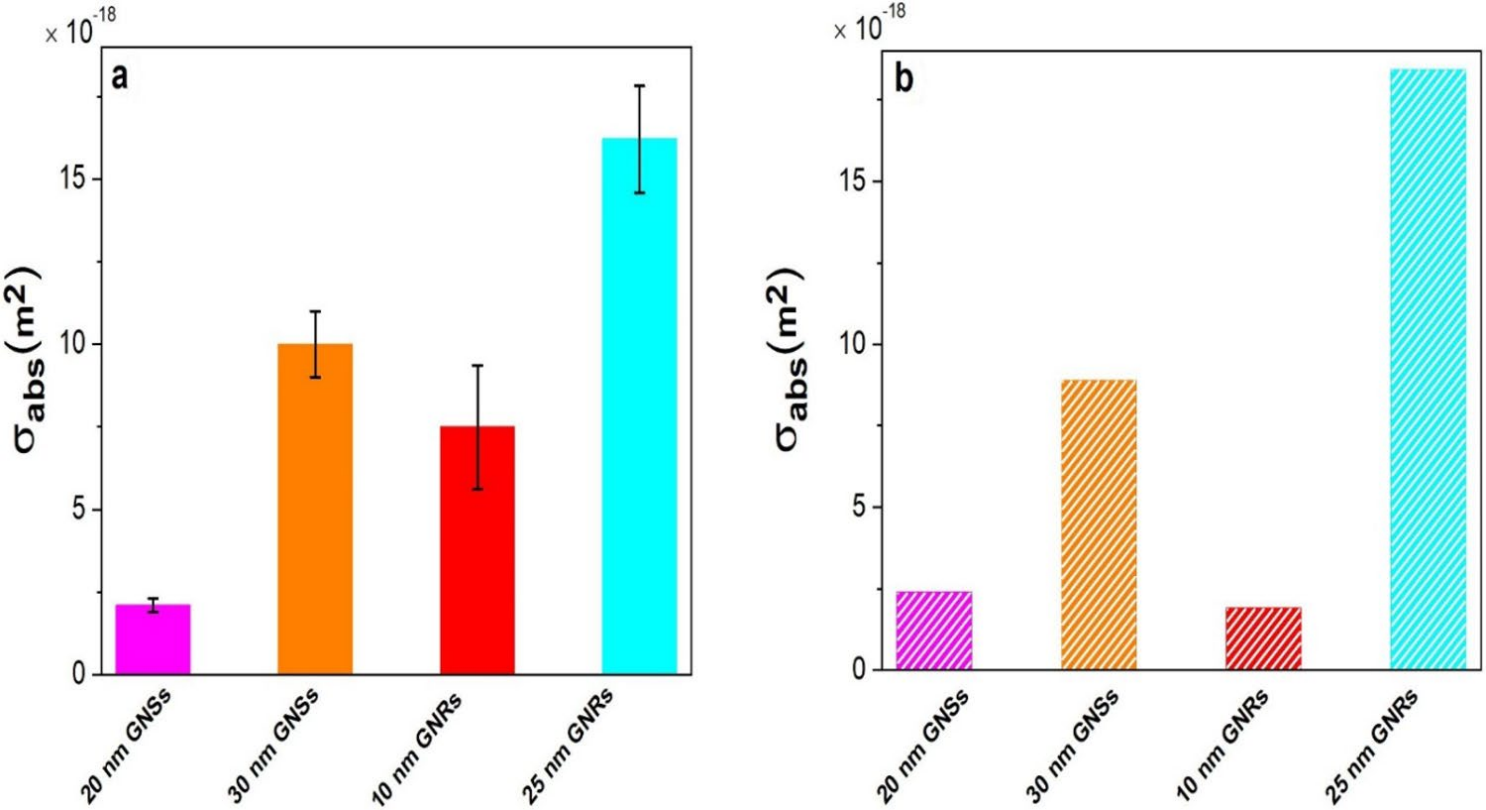
(**a**) Experimental ${\sigma }_{abs}$ (based on thermodynamic theory) and (**b**) theoretical ${\sigma }_{abs}$ (based on the Mie theory) for 20 nm and 30 nm GNSs, and 25 nm × 60 nm and 10 nm × 41 nm GNRs, wherein the error bars represent the standard deviation of triplicate measurements.

In GNRs of two different sizes of 10 nm × 41 nm and 25 nm × 60 nm, the SPR band is split into (i) transverse and (ii) longitudinal modes of oscillation of the surface plasmons^5,6^. In the extinction spectra Fig. 1b, the first mode lies at 524 nm of a rather weak band in both the samples, while the second band is displaced in a strong band at 808 nm in the first sample, which is blue shifted at 650 nm on a markedly smaller aspect ratio 2.4 in the other sample over that of 4.1 in the first sample. The first sample (R_eq_ = 9.16 nm) exhibits δT_g_ = 1.7 × $10^{ - 6}$ °C, ${\text{with}} \sigma_{abs}$ = (7.49 $\pm$ 1.252) × $10^{ - 18}$ ${\text{m}}^{2}$, which is promoted to 3.2 × $10^{ - 6}$ °C on an increased R_eq_ = 19.2 nm by a factor of 2.1, with a proportionally enhanced $\sigma_{abs}$ = (16.2 $\pm$ 1.676) × $10^{ - 18}$ ${\text{m}}^{2}$ in the other sample. Thus, R_eq_ plays a critical role in harvesting δT_g_ in correlation to ${\text{the }}\sigma_{abs} {\text{value }}$ of GNRs. While the DDA is widely used for calculating $\sigma_{abs}$ for an elongated material^7,8^, here we were able to calculate it for GNRs using R_eq_ based on the Mie theory, i.e. quite compatible to the measured value in Fig. 4. A ${\text{value of }}\sigma_{{{\text{abs}}}}$ = 1.8 × $10^{ - 18}$ ${\text{m}}^{2}$ is thus calculated against a measured (7.49 $\pm$ 1.254) × $10^{ - 18}$ ${\text{m}}^{2}$ value for 10 nm × 41 nm GNRs, while $\sigma_{abs} =$ 18.43 × $10^{ - 18}$ ${\text{m}}^{2}$ compared to the measured (16.2 $\pm$ 1.676) × $10^{ - 18}$ ${\text{m}}^{2} {\text{one }}$ for 25 nm × 60 nm GNRs, as given in Tables S3 and S4. Together, these results confirm that R_eq_ and β are two very important parameters to tailor and experimentally determine $\sigma_{abs}$ for GNRs.

Uniquely, GNUs, which contain spikes, uneven bumps, and thorns at the edges in the form of a core–shell nanostructure, duly trigger the electromagnetic fields at the surface leading to a red shift in the SPR band in a broader band of a multiple green-to-red color over 480–850 nm wavelengths as seen in Fig. 1c in comparison to those in the GNSs, useful for solar-energy, biological cells and other applications^33,34^. δT_g_ ≅ 46.5 × $10^{ - 5}$ °C, as found in a sample of 80 nm GNUs, with $\sigma_{abs}$ = (25.5 $\pm$ 4.089)$\times 10^{ - 16}$ ${\text{m}}^{2}$, is markedly enhanced to 248.5 × $10^{ - 5}$ °C in that of 100 nm GNUs of a concomitantly enhanced $\sigma_{abs}$ ≅ (18.4.0 $\pm 1.216$) × $10^{ - 15}$ ${\text{m}}^{2}$. Evidently, it is the unique surface that effectively enhances the functionalized charger carriers which, in turn, enhances the $\sigma_{abs}$ and the δT_g_ in the GNUs in comparison to those in the smooth, spherical samples.

## Conclusions

We demonstrate an analytical method for measuring the $\sigma_{abs}$ value in terms of an induced temperature change of nanogold in a colloidal suspension (upon laser irradiation), based on the heat diffusion theory and energy balance of the system. An induced temperature in this experiment depends on primary factors of size, morphology, surface functionality of the nanogold, its exchange interaction with the surrounding, and the irradiation source of a given power density. The measured and calculated $\sigma_{abs}$ values match closely for the GNS samples. Furthermore, when considering the SPR properties of light-absorption in NIR region of nanogold, the surface functionalization of 20 nm GNSs significantly improves the $\sigma_{abs}$ value by a factor of more than 10. This method can be used to estimate the $\sigma_{abs}$ of nanogold of complex shapes, such as nanorods and nanourchins. The results substantiate the nontrivial fact that GNUs promote the optical and thermal properties over GNSs and GNRs in an account of unique features of surface charge carriers. The results confirm that the optical cross section for a variety of nanomaterials can be determined by measuring the temperature change of the particles during laser irradiation and that this methodology can be extended to a variety of other materials. This approach can help develop our understanding of thermal properties of surface plasmonics by studying dynamics of laser induced heating of surface-charge-carriers in nanogold dispersed in a nanofluid in different functional structures.

## Experimental methods

### Chemical products

The samples of 10 nm × 41 nm and 25 nm × 60 nm GNRs, and 80 nm and 100 nm GNUs were purchased from Sigma-Aldrich (St. Louis, MO). 20 and 30 nm GNSs were purchased from Ted Pella (Redding, CA). GNPs functionalized with an IR 808 dye conjugate (AuNPCs) were chemically synthesized and supplied by Lahjavida (Colorado Springs, CO).

### Experimental setup

**Figure 5:**
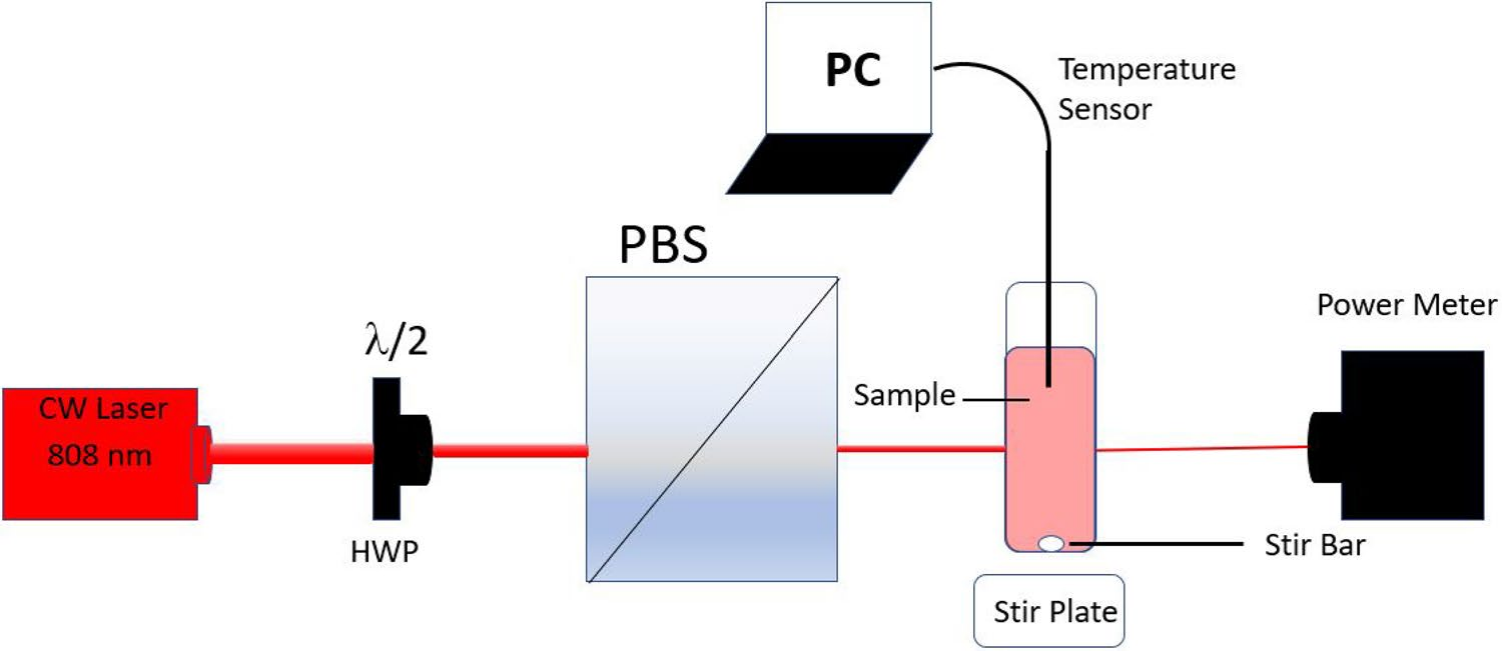
Experimental schematic for measuring temperature elevation in a nanocolloidal sample; HWP : a half-wave plate, PBS: a beam splitter, and PC: a computer.

The colloidal suspensions of nanogold (3 mL volume) were placed in a standard glass cuvette (VWR, Aurora, CO 80011), whose temperature was measured by using a thermocouple. All the solutions were studied at room temperature (23 °C) in which any induced temperature was recorded over 25 min of a CW NIR laser (808 nm) exposure, as shown in the schematic in Fig. 5. The temperature sensor was placed in the solution and not in the beam path as the scattering could lead to a non-zero response when aligning the beam, as seen in Fig. 5. A magnetic stir bar and plate, set to 300 rpm, were used to achieve uniform distribution of temperature, and a glass cuvette was used to avoid the reflection of the laser. While the cuvettes were not sealed, there was no solvent evaporation observed during the experimental measurements. The optical density (OD) of all solutions used were measured and normalized at 1. The NIR CW red laser beam of 808 nm wavelength and 4 W output power was used to determine the temperature profile of the samples. The laser power and the transmitted power were measured at a focused point by a digital power meter (PM100D, Thorlabs, Dachau, Germany). We used a half-wave plate (HWP) and a polarizing beam splitter (PBS) to adjust the power density of the beam over the sample.

### Instrumentation

The extinction spectra of nanogold colloids were measured using a UV–VIS absorption spectrophotometer (Lambda 1050, PerkinElmer). A CW red laser of 4 W power was purchased from LaserLand (Wuhan Besram Technology, Hubei, China). The temperature was recorded using a temperature sensor (PS-2146, Pasco, Roseville, CA, US) in conjunction with PASCO Capstone software.

## Supplementary information

Supplementary Information.
