# Supplementary material for: Absorption cross section of gold nanoparticles based on NIR laser heating and thermodynamic calculations
Source: Sci Rep. 2020 Nov 2;10:18790. doi: 10.1038/s41598-020-75895-9 (PMC7606525; doi:10.1038/s41598-020-75895-9)
Supplement: Supplementary file 1 — Supplementary Information. [file 41598_2020_75895_MOESM1_ESM.pdf]

# Absorption Cross Section of Gold Nanoparticles based on NIR Laser Heating and Thermodynamic Calculations


Mazen Alrahili[1,3] *, Viktoriia Savchuk[1], Kelly McNear[2], and Anatoliy Pinchuk [1,2]

[1]Department of Physics and Energy Science, University of Colorado Colorado Springs, 1420 Austin Bluffs Parkway, Colorado Springs, Colorado 80918, USA

[2]UCCS BioFrontiers Center, University of Colorado Colorado Springs, 1420 Austin Bluffs Parkway, Colorado Springs, Colorado 80918, USA

[3]Physics Department, School of Science, Taibah University, Janadah Bin Umayyah Road, Medina 42353, Saudi Arabia

## Corresponding Author

Mazen Alrahili

E-mail: mrahili@taibahu.edu.sa

**Table S1: The SPR, mass, and concentration of gold nanomaterials (GNPs).**

| Gold Nanomaterials | SPR Wavelength (nm) | Mass of 1 Particle (kg) | Concentration (NPs/mL) |
|---|---|---|---|
| 20 nm GNSs | 520 | $8.08\times10^{-20}$ | $7.00\times10^{11}$ |
| 30 nm GNSs | 520 | $2.73\times10^{-19}$ | $2.00\times10^{11}$ |
| AuNPC | 520 | $8.08\times10^{-20}$ | $7.00\times10^{11}$ |
| 25 nm × 60 nm GNRs | 650 | $6.50\times10^{-20}$ | $7.20\times10^{11}$ |
| 10 nm × 41 nm GNRs | 808 | $9.80\times10^{-21}$ | $5.72\times10^{12}$ |
| 80 nm GNUs | 620 | $5.20\times10^{-18}$ | $7.82\times10^{9}$ |
| 100 nm GNUs | 680 | $1.02\times10^{-17}$ | $3.84\times10^{9}$ |

Gold nanomaterials specifications can be obtained from Sigma Aldrich and Ted Pella:

(https://www.sigmaaldrich.com and https://www.tedpella.com or https://www.bbisolutions.com)

**Table S2: calculation of the mass of GNPs and the solution and calculation of the temperature distribution by a single GNP for each size and morphology.**

| GNPs shapes | $m_g$(Kg) | $m_S$(Kg) | $\delta T_g = \frac{m_S C_S}{m_{Au} C_{Au}} \delta T_S$ |
|---|---|---|---|
| 20 nm GNSs | $1.69\times10^{-7}$ | 0.003 | $\delta T_g = 5.7 \times 10^5 \, \delta T_S$ |
| 30 nm GNSs | $1.64\times10^{-7}$ | 0.003 | $\delta T_g = 5.9 \times 10^5 \, \delta T_S$ |
| AuNPC | $1.69\times10^{-7}$ | 0.003 | $\delta T_g = 5.7 \times 10^5 \, \delta T_S$ |
| 25 nm × 60 nm GNRs | $1.40\times10^{-7}$ | 0.003 | $\delta T_g = 6.9 \times 10^5 \, \delta T_S$ |
| 10 nm × 41 nm GNRs | $1.68\times10^{-7}$ | 0.003 | $\delta T_g = 6.2 \times 10^5 \, \delta T_S$ |
| 80 nm GNUs | $1.22\times10^{-7}$ | 0.003 | $\delta T_g = 7.9 \times 10^5 \, \delta T_S$ |
| 100 nm GNUs | $1.12\times10^{-7}$ | 0.003 | $\delta T_g = 8.6 \times 10^5 \, \delta T_S$ |

To calculate the mass of Au:

$$m_g\text{(kg)} = \text{Concentration (NPs/mL)} \times \text{Mass of 1 Particle (kg)} \times \text{Volume (3 mL)}$$

**Table S3. Summary of the measured experimental values of radius and the equivalent radius, the slopes, and the absorption cross sections.**

| GNPs Shapes | R or $R_{eq}$(nm) | β | Slope values (°C*$m^2$/W) | Absorption Cross Section $\sigma_{abs}(m^2)$ |
|---|---|---|---|---|
| 20 nm GNSs | 10 | 1 | $(2.81 \pm 0.423) \times 10^{-11}$ | $(2.1 \pm 0.316) \times 10^{-18}$ |
| 30 nm GNSs | 15 | 1 | $(8.85 \pm 0.829) \times 10^{-11}$ | $(10.0 \pm 0.937) \times 10^{-18}$ |
| AuNPC | 10 | 1 | $(14.87 \pm 1.127) \times 10^{-11}$ | $(11.2 \pm 0.847) \times 10^{-16}$ |
| 25 nm × 60 nm GNRs | 19.16 | 1.74 | $(6.34 \pm 0.656) \times 10^{-11}$ | $(16.20 \pm 1.676) \times 10^{-18}$ |
| 10 nm × 41 nm GNRs | 9.16 | 2.92 | $(3.72 \pm 0.623) \times 10^{-11}$ | $(7.49 \pm 1.254) \times 10^{-18}$ |
| 80 nm GNUs | 40 | 1 | $(8.46 \pm 1.355) \times 10^{-9}$ | $(25.5 \pm 4.084) \times 10^{-16}$ |
| 100 nm GNUs | 50 | 1 | $(48.98 \pm 3.237) \times 10^{-9}$ | $(18.4 \pm 1.216) \times 10^{-15}$ |

**Table S4. Summary of the Calculated Absorption Efficiency and Absorption Cross Section By Mie Theory.**

| GNPs Shapes | R or $R_{eq}$(nm) | Absorption Efficiency | Absorption Cross-Section $\sigma_{abs}(m^2)$ |
|---|---|---|---|
| 20 nm GNSs | 10 | 0.0077 | $2.4 \times 10^{-18}$ |
| 30 nm GNSs | 15 | 0.012 | $8.5 \times 10^{-18}$ |
| 25 nm × 60 nm GNRs | 19.15 | 0.016 | $18.43 \times 10^{-18}$ |
| 10 nm × 41 nm GNRs | 9.16 | 0.007 | $1.8 \times 10^{-18}$ |

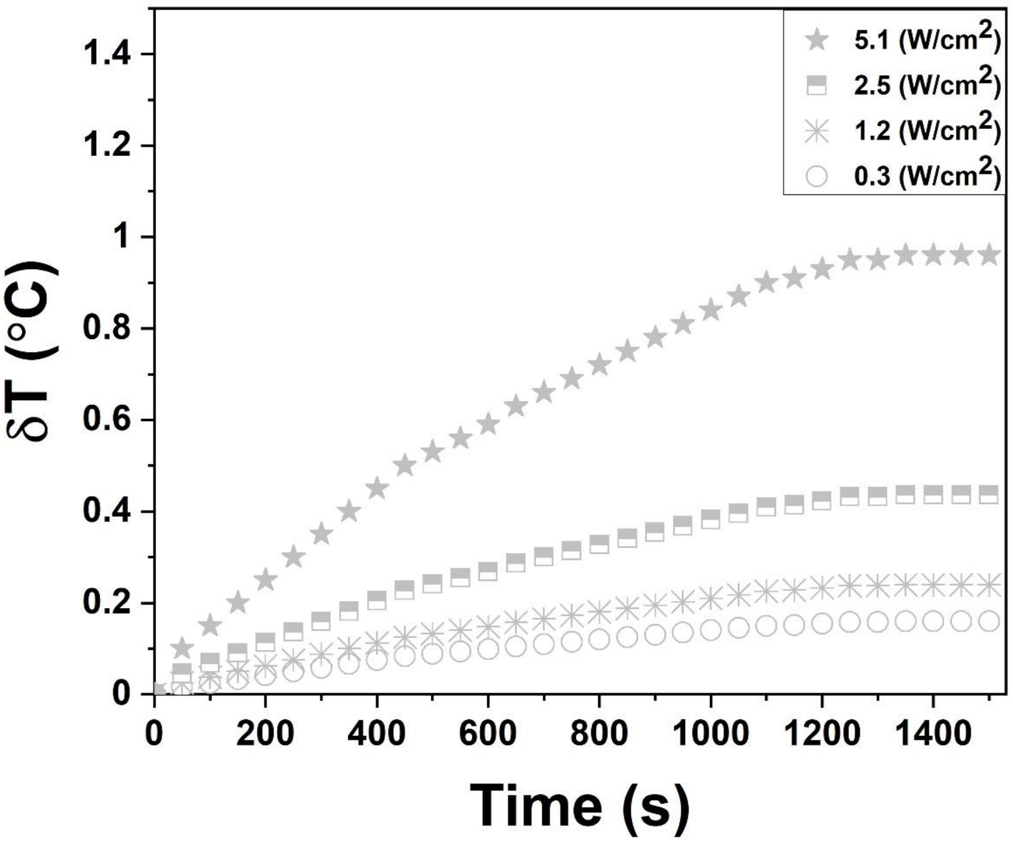


**Figure S1.** Temperature elevation of the solvent of the GNPs as a function of time in different power densities. The solvent is Ultra-Pure Water (UPW)

**Absorption Cross Section By Mie Theory**

The optical properties of GNSs and GNRs were quantified in terms of their calculated absorption efficiencies by Mie theory for the homogenous sphere. The required parameters for the calculations are the

radius and the refractive index of the surrounding medium, which is n=1.33 for water, as well as the excitation wavelength of 808 nm.[1]

$$Q_{sca} = \frac{2}{(^{2\pi r}/_{\lambda})^2}\sum_{l=1}^{\infty}(2l+1)(|a_l|^2 + |b_l|^2) \tag{15}$$

$$Q_{ext} = \frac{2}{(^{2\pi r}/_{\lambda})^2}\sum_{l=1}^{\infty}(2l+1)\mathrm{Re}(a_l + b_l) \tag{16}$$

$$Q_{abs}=Q_{ext}-Q_{sca} \tag{17}$$

Here, $a_l$ and $b_l$ are the expansion coefficients in terms of Ricatti-Bessel functions( $\psi(x)$ and $\eta\,(x)$ ) defined as follows,

$$a_l=\frac{m\psi\,(mx)\psi'(x)-\psi'(mx)\psi(x)}{m\psi\,(mx)\,\eta'\,(x)-\psi'(mx)\,\eta\,(x)} \tag{18}$$

$$b_l = \frac{\psi\,(mx)\psi'(x)-\psi'(mx)\psi(x)}{\psi\,(mx)\,\eta'\,(x)-\psi'(mx)\,\eta\,(x)} \tag{19}$$

Here m = $\frac{n}{n_m}$, where $n$ is a complex refractive index and $n_m$ is a real refractive index of the surrounding medium and $x$=kR, where k is the wavenumber and R is the radius of the particle. After calculating the scattering and extinction efficiencies, we can determine the absorption efficiency by using eq 20. Fig. S2 (a-b) shows the calculated spectra of the absorption efficiency, $Q_{abs}$, for 20 nm and 30 nm GNS, 25 nm × 60 nm and 10 nm × 41 nm GNRs, and 80 nm and 100 nm GNUs. The dimensionless efficiencies can be converted to the $\sigma_{abs}$ per unit area by the following expression[2]

$$\sigma_{abs} = Q_{abs}\,\pi r^2 \tag{20}$$

where $Q_{abs}$ can be obtained from Fig. S2 (a-b) at the wavelength 808 nm and r is the radius or the equivalent radius. Table S4 of the supporting information summarizes the calculated absorption efficiencies and the

absorption cross sections for 20 nm and 30 nm GNSs, 25 nm × 60 nm, 10 nm × 41 nm and GNRs, and 80 nm and 100 nm GNUs. These experimental values of the $\sigma_{abs}$ are evidenced by our experimental methodology and applying these results into the heat transfer theory and energy balance of the system.

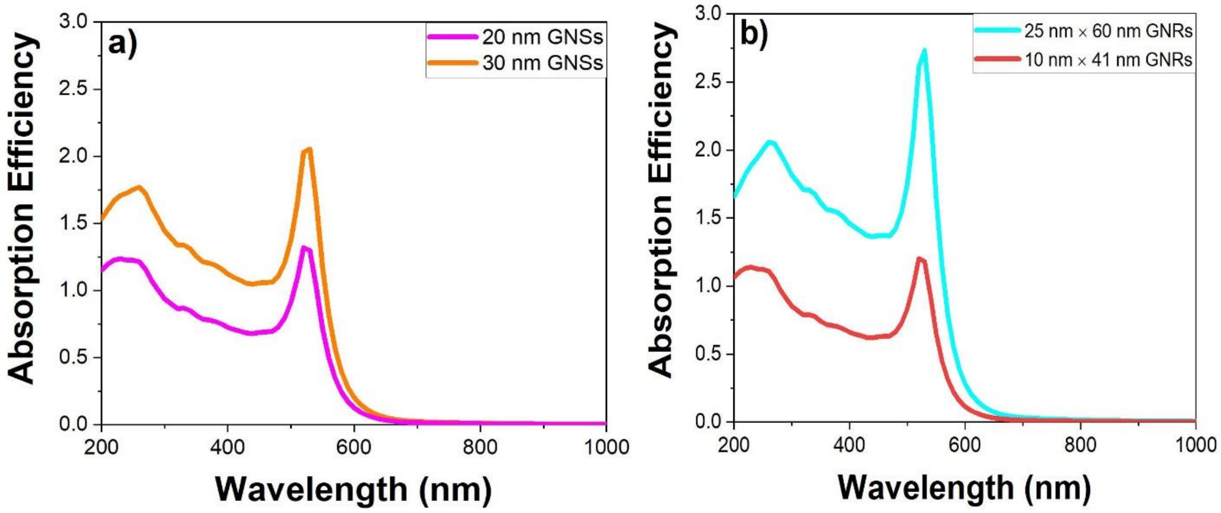


**Figure S2.** Calculated spectra of the dimensionless absorption efficiency of 20 and 30 nm GNS (a) and 25 nm × 60 nm and 10 nm × 41 nm GNR (b) by Mie theory. The calculated absorption efficiency of GNRs based on the equivalent radius.

References


1 Jain, P. K., Lee, K. S., El-Sayed, I. H. & El-Sayed, M. A. Calculated absorption and scattering properties of gold nanoparticles of different size, shape, and composition: Applications in biological imaging and biomedicine. *J. Phys. Chem. B* **110**, 7238–7248 (2006).

2 Pérez-Juste, J., Pastoriza-Santos, I., Liz-Marzán, L. M. & Mulvaney, P. Gold nanorods: Synthesis, characterization and applications. *Coordination Chemistry Reviews* **249**, 1870–1901 (2005).
